# Promotion of cancer cell stemness by Ras

**DOI:** 10.1042/BST20200964

**Published:** 2021-02-05

**Authors:** Rohan Chippalkatti, Daniel Abankwa

**Affiliations:** Cancer Cell Biology and Drug Discovery Group, Department of Life Sciences and Medicine, University of Luxembourg, 4362, Esch-sur-Alzette, Luxembourg

**Keywords:** cancer stem cells, centrosomes, cilia, KRAS, trafficking

## Abstract

Cancer stem cells (CSC) may be the most relevant and elusive cancer cell population, as they have the exquisite ability to seed new tumors. It is plausible, that highly mutated cancer genes, such as *KRAS*, are functionally associated with processes contributing to the emergence of stemness traits. In this review, we will summarize the evidence for a stemness driving activity of oncogenic Ras. This activity appears to differ by Ras isoform, with the highly mutated *KRAS* having a particularly profound impact. Next to established stemness pathways such as Wnt and Hedgehog (Hh), the precise, cell cycle dependent orchestration of the MAPK-pathway appears to relay Ras activation in this context. We will examine how non-canonical activities of K-Ras4B (hereafter K-Ras) could be enabled by its trafficking chaperones calmodulin and PDE6D/PDEδ. Both dynamically localize to the cellular machinery that is intimately linked to cell fate decisions, such as the primary cilium and the centrosome. Thus, it can be speculated that oncogenic K-Ras disrupts fundamental polarized signaling and asymmetric apportioning processes that are necessary during cell differentiation.

## Introduction

**Figure 1.**
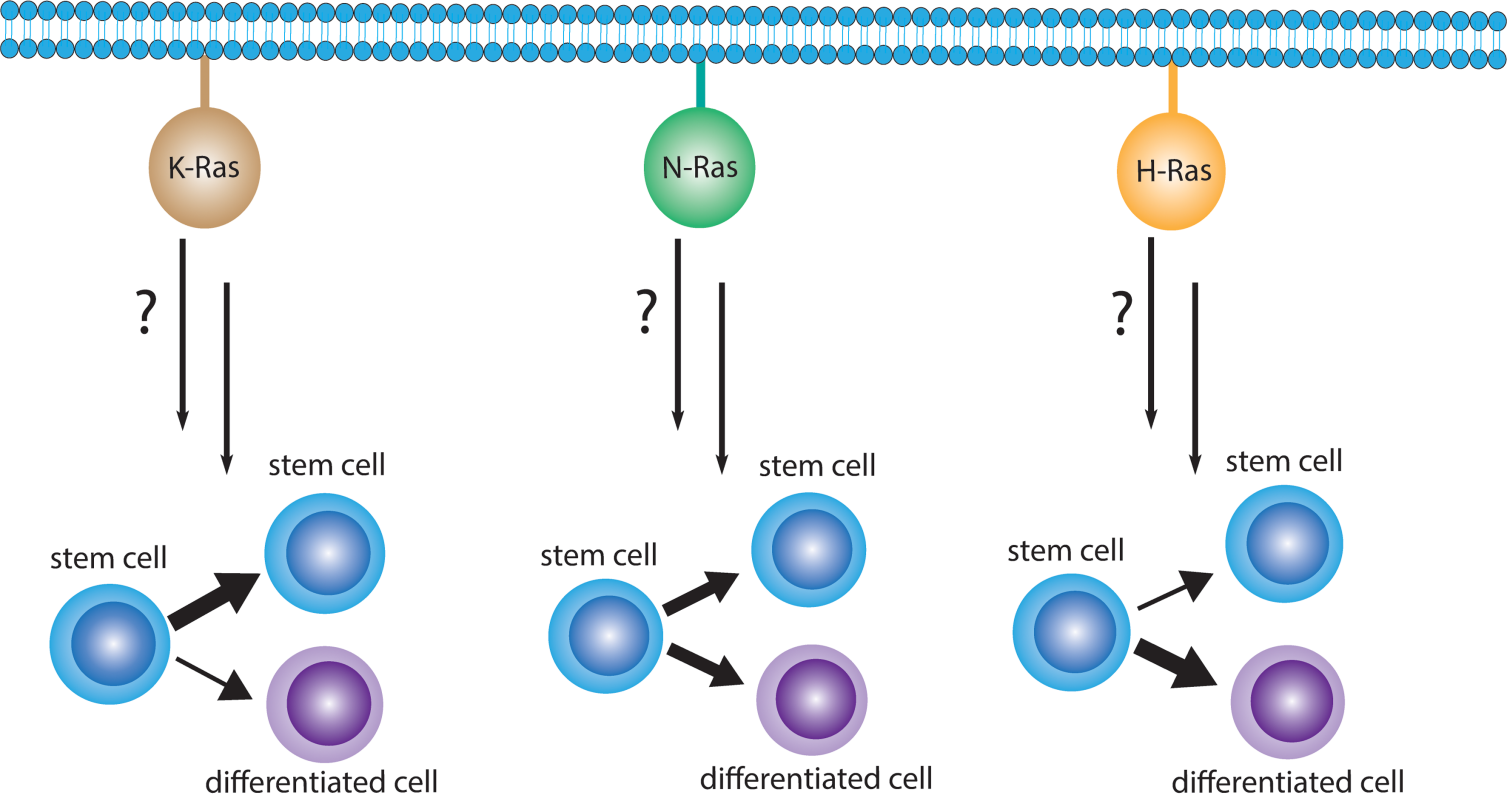
The canonical Ras proteins appear to have isoform specific potential to drive stemness. In the few direct comparisons that have been studied, K-Ras has the highest potential to induce stemness traits. N-Ras appears to be neutral or have a bimodal activity. At the other end of the spectrum, H-Ras may rather drive differentiation, while it is in some context also able to support stemness.

During the morphogenesis of normal tissues, the balance between stem cell self-renewal and differentiation is regulated by symmetric and asymmetric cell divisions [1]. Certain features of these ontogenetic processes are hijacked in cancer by CSC, which are conceptualized as the cells that initiate a tumor, thus fuelling intra-tumoral clonal diversity and seeding metastasis [2]. Similar to their normal stem cell counterparts they appear to be endowed with a self-renewal capacity. Their quiescence, render CSC comparatively insensitive to classical anti-proliferative drugs [3]. Therefore, specific CSC targeting drugs were sought in recent years, which typically emerged from phenotypic screens [4,5]. However, most of these approaches delivered compounds with divergent targets. Often these targets were not obviously associated with stemness, unless inhibitors of stemness pathways such as Wnt and Hh were assessed [6]. One campaign stumbled across the fact that several experimental CSC drugs selectively affect K-Ras, but not H-Ras [7]. This was in line with data by the McCormick group showing that mutant K-Ras, but not H-Ras has exquisite potential to instruct stemness traits in cancer cells [8]. Earlier, the Settleman group made similar observations in the F9 stem cell model, showing that oncogenic K-Ras promotes proliferative expansion of the stem cells, and N-Ras adopts a neutral role, while H-Ras induces differentiation [9] (Figure 1). This Ras-isoform dependent propensity to promote stemness strikingly correlates with the mutation frequency of *RAS* genes, with *KRAS, NRAS* and *HRAS* being mutated in 75%, 17% and 7% of *RAS*-mutant human cancers [10]. In this review, we will examine the evidence for Ras proteins being involved with the emergence of stemness traits in cancer cells.

## Evidence for CSC in RAS driven cancers

*HRAS* is the most frequently mutated (5.1%) *RAS* gene in head and neck squamous cell carcinomas [10]. Recently, the farnesyltransferase inhibitor tipifarnib has been reintroduced for treatment of *HRAS* mutant cancers [11]. While tipifarnib can affect several farnesylated proteins, it is interesting to note that it down-regulated CSC marker CD44 [11]. Expression of markers like CD44, CD133/prominin-1 or of classical pluripotency markers such as OCT4, SOX2 and NANOG are used to characterize stemness in cancer cells [12–15]. Functionally, stemness is frequently assessed in clonogenic 3D spheroid assays under serum-free conditions to analyze the self-renewal potential [16]. Consistently, serum depletion induces CSC-like properties in *HRAS-G12V* transformed mouse embryonic fibroblasts, which then displayed prominin-1 and elevated levels of OCT4 [17].

Some drug treatments may trigger intrinsic feedback loops, which turn into stemness promoting resistance mechanisms. Rapamycin increases the expression of galectin-1, a nanocluster scaffold of GTP-H-Ras [18,19]. Active Ras nanocluster are signaling platforms of Ras, Raf-effectors and other modulators of Ras signaling [20]. In addition to increasing Ras-MAPK-signaling, galectin-1 promoted the expression of the CSC marker CD44 and increased sphere formation in a *HRAS*- but not in a *KRAS*-mutant breast cancer cell line [19]. A related study established that stimulation of mTORC1 with amino acids increases oncogenic H-Ras- but decreases K-Ras-nanoclustering via a phosphatidic acid-dependent mechanism downstream of SREBP1, with attendant consequences for spheroid growth of *HRAS* and *KRAS* mutant cancer cells [21]. Similar to its intermediate activity in stem cells, oncogenic N-Ras appears to drive cancer cell stemness in a bi-modal fashion, increasing the self-renewal capacity in one subset of hematopoietic stem cells, while increasing cell division in another subset [22].

Activating *KRAS* mutations are most frequently observed in pancreatic cancer (88%) [10]. Several pancreatic cancer cell lines (e.g. PANC1, SW1990 and Patu8988) express prominin-1, OCT4, SOX2, NANOG and show increased sphere-forming capacity in a K-Ras dependent manner [23]. Activating mutations in *KRAS* furthermore correlated with a higher proportion of DLD1 colon cancer cells staining positively for CD44 and prominin-1, as well as for typical pluripotency markers, while xenografting of these cells demonstrated increased tumor propagation capacity [24]. Similarly, tumorospheres of *KRAS*-mutant lung adenocarcinoma cell lines, A549, H358, H23, up-regulated prominin-1, OCT4 and NANOG and showed enhanced self-renewal capacity [25].

It will be interesting to examine the CSC-specific effect of potent K-Ras inhibition, using recent G12C-specific covalent inhibitors as tools, given that these compounds reduce 3D spheroid growth more *KRAS*-selectively than 2D growth [26].

## K-Ras and stemness signaling pathways

Oncogenic K-Ras can increase classical stemness signaling pathways, such as Hh-signaling in pancreatic ductal cells, which normally lack sonic hedgehog [27]. Likewise, K-Ras can up-regulate components of the Wnt/β-catenin-pathway [28]. Both the Wnt/β-catenin- and the Hh-pathways operate at the primary cilium (PC), an important sensory organelle with critical roles in stem cell self-renewal [29–32]. MAPK-signaling was found to be initiated from within the PC in NIH-3T3 fibroblasts [33] and treatment with MAPK inhibitor reduced PC length in MDCK cells [34]. Intriguingly, subcellular trafficking pathways of K-Ras imply its association with the PC [35,36].

The K-Ras stemness mechanism by Wang et al. suggested that the ability of K-Ras, but not H-Ras, to bind and sequester calmodulin (CaM), suppresses non-canonical Wnt-signaling. A K-RasG12V-dependent decrease in Ca^2+^/calmodulin-dependent kinase II (CaMKII) activity, then downmodulates the expression of the Wnt-ligand Fzd8 [8]. Importantly, the interaction between K-Ras and CaM is suppressed by phosphorylation of Ser181 in the C-terminal membrane anchoring region of K-Ras, while conversely binding of CaM prevents phosphorylation [37,38]. AMPK signaling, which exhibits a complex interplay with MAPK signaling [39], promotes Ser181 phosphorylation through PGK2 and could thus disrupt the K-Ras/CaM interaction [40]. Furthermore, drugs that have the potential to increase Ser181-phosphorylation, such as the atypical PKC agonist prostratin, as well as CaM inhibitors have been shown to reduce CSC properties [7,8].

**Figure 2.**
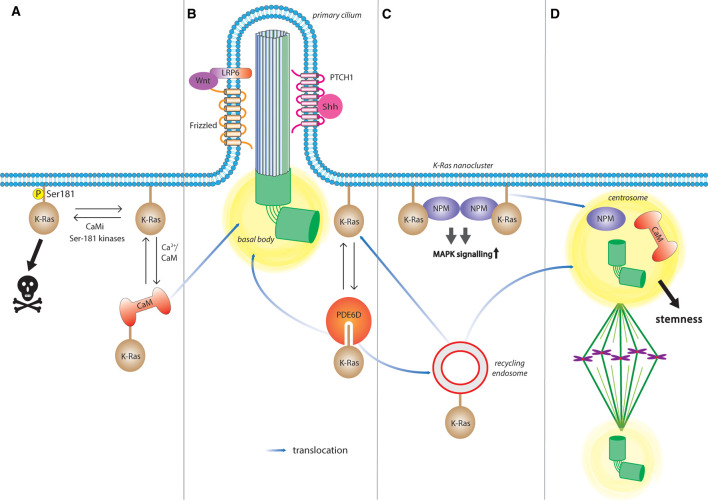
Pathways and interactions regulating K-Ras driven stemness. (**A**) CaM enhances K-Ras driven stemness through inhibition of non-canonical Wnt-signaling. CaM inhibitors (CaMi) and several kinases facilitate Ser-181 phosphorylation, thus promoting apoptosis. Conversely, CaM prevents phosphorylation of K-Ras at Ser-181 and enables K-Ras translocation. (**B**) Given the dynamic localization of CaM to several mitotic structures and the plasma membrane during mitosis, K-Ras could access these structures while chaperoned by CaM. Similarly, PDE6D/ PDEδ solubilizes K-Ras from membranes and unloads it at the recycling endosome for transport. Both PDE6D and CaM could therefore position K-Ras on the basal body of the primary cilium, which harbors several components of stemness signaling, such as those of the canonical Wnt- and Hh-pathways. (**C**) In the plasma membrane, K-Ras is organized into nanoclusters, signaling centres that enhance MAPK-signaling. Nanoclustering can be stabilized by NPM1, which is known to control centrosome duplication. (**D**) The (mother) centrosome derived from the mother centriole of the basal body of the PC is typically retained by the stem cell during asymmetric division. CaM and PDE6D facilitate the distribution of K-Ras during the cell cycle. Moreover, unloading of mitotic cargoes at the centrosome by the recycling endosome may imply that in particular K-Ras can impact on these stemness processes.

Ca^2+^/CaM is a trafficking chaperone of K-Ras, which can bind up to two K-Ras proteins via their C-terminal farnesyl-moiety [41–43]. Another trafficking chaperone of K-Ras is PDE6D (also known as PDEδ), which also sequesters the C-terminal farnesyl, and ultimately facilitates plasma membrane trafficking of K-Ras [35]. It essentially enhances the diffusion rate of K-Ras between cellular membranes and to the recycling endosome, from where K-Ras is actively transported back to the plasma membrane [44]. Plasma membrane anchorage is necessary for K-Ras to engage its canonical effectors, hence its disruption effectively reduces its oncogenic activity, such as demonstrated by various PDE6D inhibitors [45–47]. Consistently, inhibition of CaM could have similar effects on K-Ras trafficking, justifying renewed interest in screening for CaM inhibitors [48] (Figure 2A).

Of note, Ser181 phosphorylation changes also the specific lateral organization of K-Ras from a preferred co-distribution with phosphatidylserine (PS) to phosphatidyl-inositol-4,5-bisphosphate (PIP_2_) [49]. This could alter its selectivity for certain effectors that employ these lipids for co-incidence detection and/or change nanoclustering of K-Ras. Therefore, the precise distribution of acidic lipids across polarized cells, could significantly impact on K-Ras-signaling output.

## Ras-MAPK-signaling in stem cell priming and in developmental disorders

While we are mostly concerned with cancer when discussing CSC, we may learn about the particular roles of Ras proteins from its normal function in stem cells and during development. Knockout studies support a requirement of *KRAS* for embryonal survival as compared with the other two *RAS* genes, which can be deleted without compromising viability [50]. This indicates some redundancy in the roles of the four cancer-associated Ras isoforms (splice isoforms K-Ras4A and K-Ras4B, N-Ras and H-Ras), which is further complicated by the fact that all Ras are capable of engaging the same set of effectors, notably of the MAPK- and PI3K/mTORC1-pathways [51]. It is currently not fully understood, how this promiscuous setting can give rise to biological specificity that would instruct distinct stemness potentials. However, fundamental differences in the membrane organization of Ras isoforms and the precise utilization of certain effector paralogs, plus additional specific modulatory protein interactions are emerging as key in this context [20,52–54].

A fundamental role of Ras-MAPK-signaling in stem cells is suggested by the fact that embryonic stem cells undergo a Ras-MAPK dependent transformation from the naïve to the primed pluripotent state, followed by their proliferation and differentiation during embryogenesis [55]. In line with this, isogenic iPSC derived from patients with *KRAS–G13C* mutations retained a higher level of stemness markers in G13C/wt cells and had a larger OCT4^+^ population than the wt/wt cells [56]. While similar observations for iPSC were made with N-Ras [57], again only *KRAS–G12D*, but not the other two Ras isoforms, was able to increase the neural stem cell pool when expressed in the mouse ventricular zone [58]. In this context, it may be interesting to understand, whether distinct oncogenic *RAS* alleles have a specific CSC promoting potential, given that they have different tumorigenicity [59]. A significant involvement of MAPK-signaling is furthermore supported by the fact that also BRAF-V600E promotes stemness traits [60], while MEK inhibitors broadly block stemness [9,24,56].

The diverse Ras-pathways that are relevant for stemness may be gleaned from E-Ras, which is specifically expressed in embryonic stem cells (ESC) and few adult cells, such as the hepatic stellate cells (HSC), a liver resident stem cell population [61]. Expression of E-Ras can enhance mouse iPSC induction via the mTORC2-pathway and repression of FOXO1 [62,63]. This Ras isoform is naturally GAP-insensitive, i.e. it is constitutively active and hence transcriptionally regulated [64]. In quiescent HSC of a healthy liver, E-Ras is expressed and stimulates e.g. the mTORC2-Akt- and RASSF5-HIPPO-pathways, thus repressing FOXO1 and YAP, respectively. Upon liver injury HSC become activated, which is accompanied by E-Ras down-regulation and a signaling shift to the MAPK-pathway by up-regulation of other Ras isoforms (M-Ras, R-Ras, RalA, Rap2A) [64,65]. Altogether, these data suggest that certain signaling pathways downstream of specific Ras isoforms, such as K-Ras and E-Ras, are important for the induction of cellular stemness states.

RASopathies are caused by germline mutations in Ras-MAPK-pathway genes, thus introducing developmental imbalances at the earliest stages. Affected individuals broadly display skeletal malformations, cardiac defects, various degrees of mental retardation and an increased risk for certain types of cancer [66]. The most frequent RASopathy, neurofibromatosis type I (occurrence 1: 3000), is caused by mutations of the *NF1* tumor suppressor gene, which in mice was also linked to neural stem cell hyperproliferation [67]. *NF1* encodes neurofibromin, a Ras specific GTPase activating protein (GAP), which inactivates Ras-signaling [51]. Neurofibromin is targeted to the plasma membrane by SPRED proteins, while SPRED1 piggybacks on activated B-Raf, which delivers the complex to K-Ras membrane domains [68-70]. Intriguingly, in this mechanistic complex, three highly mutated cancer genes are involved, which may point to a particularly critical relevance of this process. Recently, the major mitosis driving kinase, CDK1, was shown to phosphorylate Ser105 on SPRED1 thus blocking its interaction with neurofibromin, suggesting an important cell cycle-dependent modulation of this interaction [71]. Together with the fact that up-regulation of SPRED proteins is associated with differentiation, this mechanism may point to a central, cell cycle and differentiation-associated function of K-Ras [72,73]. Hence, GAP-desensitizing hot spot mutations in Ras do not only promote cell cycle entry to stimulate proliferation, but in addition withdraw Ras from its inactivation during cell-cycle associated differentiation processes, such as mediated by neurofibromin-SPRED-complexes. Thus cell cycle re-entry requires concerted processes that involve not only canonical phosphorylation events by CDK1 of a wide range of targets that regulate centrosome maturation, nuclear envelope breakdown and spindle assembly during mitosis, but also CDK1-mediated licensing of K-Ras-signaling [74]. This connection is particularly interesting, considering that other CDKs, such as CDK4, cooperate with K-Ras in tumorigenesis [75,76]. With the above-mentioned regulation of the SPRED1/NF1 interaction by CDK1 during the cell cycle in mind, a continuous inhibition of CDKs may work for short bouts of cancer therapy, but not long-term for the treatment of typically younger RASopathy patients.

## K-Ras trafficking to asymmetrically inherited cellular organelles

The dynamics of the subcellular distribution of Ras in the cellular life cycle are likely to provide selective access of Ras to subcellular compartments that are associated with the cellular machinery that decides between symmetric or asymmetric divisions [44,77,78]. The decision which of the two daughter cells inherits the stemness traits during asymmetric division depends on cell polarizing cues in the niche [1]. These cues can originate for instance from apical growth factor signal input or the basal extracellular matrix, and instruct the distribution of polarity proteins, which then effect the orientation of the spindle apparatus and the ensuing apportioning of cellular organelles during cytokinesis [79].

The PC emerges from the older centrosome after cell division, and it is this older centrosome that is typically inherited by the stemness-retaining cell during asymmetric division [80,81]. One reason for the stemness retention may be that the cell with the older centrosome gives rise earlier to the PC, and thus becomes sooner sensitive to Wnt- and Hh- stemness signaling [29].

Both the PC and centrosome are visited by the recycling endosome, which is significant for spindle organization and orientation [82–84]. K-Ras is trafficking via recycling endosomes [44], while CaM dynamically localizes to the centrosome and also PC [85–87]. It is not established, whether K-Ras indeed co-localizes with these centriolar structures, however its trafficking chaperone PDE6D does [36,88]. High affinity PDE6D clients like INPP5E localize deep inside of the cilium by the concerted activity of PDE6D, its release factor Arl3 and the Arl-GEF Arl13B [36]. However, the affinity of K-Ras to PDE6D is relatively low, which may preclude transport deep into the cilium, but could place it at the base of an organelle that is central to stemness pathways and cell fate decisions [89]. Thus, the K-Ras/ CaM-stemness axis is positioned at decisive points of the cellular machinery that executes cell fate decisions. It is therefore plausible to assume that certain inhibitors (e.g. against PDE6D or CaM) exert their somewhat selective anti-stemness activity on K-Ras not just at the plasma membrane, but more so during cell division, by fundamentally affecting the inheritance of centrosome-associated stemness traits (Figure 2B).

The most prominent stem cell marker CD133/prominin-1 is a penta-span cholesterol-binding protein enriched in curved membranes, including the tip of the PC [90]. Recently, it was shown that it actively promotes stem cell self-renewal and proliferation by controlling the recruitment of PC components [31]. Moreover, prominin-1 is important for autophagy inhibition, a process that is relevant for asymmetrically inherited midbody remnants, which are prominin-1 enriched in the stemness-retaining cell [91]. Under low serum conditions, prominin-1 is in complex with HDAC6 and traffics to the pericentrosomal region, where it inhibits the autophagy initiator GABARAP. From there, prominin-1 is returning to the plasma membrane via the recycling endosome [92]. Interestingly, the promotor of prominin-1 contains binding sites for Ets, downstream of the Ras-MAPK-pathway and its expression can be increased by oncogenic Ras [93]. Considering that autophagy inhibitors have been shown to attenuate tumorigenicity in K-Ras driven pancreatic cancer and are being evaluated in clinical trials [94], it could be important to understand, whether these compounds act by affecting fundamental stemness processes, such as midbody retention.

## Potential impact of K-Ras on the mode of cell division

Ras impacts on cell cycle processes from multiple angles, such as for example by signaling for centrosome amplification [95]. The classical focus on Ras-signaling originates from its role to mediate mitogenic stimuli, thus driving cell cycle progression and proliferation. This focus is in line with standard 2D cancer cell proliferation assays. However, cancer is as much a disease of aberrant differentiation [96], and within the CSC-concept a disease of erroneous cell fate decisions that take place during the cell cycle.

Nucleophosmin 1 (NPM1) together with nucleolin increases K-Ras membrane binding, and NPM1 in addition nanoclustering by an as yet not fully defined mechanism [97] (Figure 2C). Like CaM, it binds less to K-RasG12V-S181D, the phosphomimetic mutant that has less stemness promoting activity [8,38,97]. Indeed, knockdown of NPM1 was shown to inhibit self-renewal in neural stem cells [98]. NPM1 localizes to the centrosome to control its duplication and has been shown previously to regulate mitotic spindle assembly [99–101]. Therefore, ciliary localization of K-Ras could position NPM1 to control centrosome duplication at the basal body. This would require access of nuclear NPM1 to K-Ras, which would also allow for the transient stabilization of K-Ras nanocluster. In line with this, we found that several nuclear export inhibitors of the leptomycin B family selectively block K-Ras nanoclustering and stemness traits [7].

Furthermore, K-Ras distribution may in this context be regulated by CaM, which highly dynamically distributes to the cell cortex and centrosome [85]. Alternatively, given that CaM is a bivalent binder, it could effectively act as a scaffold to up-concentrate proteins like K-Ras, while ‘holding on’ to another location, such as the centrosome [102]. Oncogenic activation of K-Ras could perturb NPM1 localization to the centrosomes, which are kept free from NPM1 after its phosphorylation by CDK1/ cyclin E between G1 and mitosis [103] (Figure 2D). This could inadvertently promote centrosome maturation, which in consequence would sabotage asymmetric cell division, as there would now effectively be two ‘mother centrosomes’ present. While this is a hypothetical scenario, mis-localization of oncogenic Ras-signaling complexes would effectively randomize their activities across the cell. Ultimately, this could lead to a fatal disruption of fundamental polarized signaling and asymmetric apportioning processes during stem cell division.

## Perspectives

- Tumor seeding during clonal evolution or metastasis is ascribed to CSC, which should position CSC in the focus of cancer therapy. However, the emergence of stemness traits in cancer cells is poorly understood.
- *KRAS* mutations are associated with aggressive tumors and K-Ras is an established drug target with emerging direct inhibitors for K-Ras-G12C. The classical focus is on Ras-MAPK-signaling driving cell proliferation. However, reports from recent years support a significant role in particular of K-Ras in driving stemness processes.
- Beyond its canonical functions, K-Ras may impact on the core cellular machinery that operates during cell fate decision-making. We know little about its activities and localization during the cell cycle and on organelles, such as the centrosome that can transmit stemness traits.

## Abbreviations

AMPK: AMP-activated protein kinase
aPKC: atypical protein kinase C
Arl13B: ADP-ribosylation factor-like protein 13B
CaM: calmodulin
CaMKII: Ca^2+^/calmodulin-dependent kinase II
CDK1: cyclin dependent kinase 1
CSC: cancer stem cell
ESC: embryonic stem cells
Fzd8: frizzled 8
GAP: GTPase activating protein
GEF: guanine nucleotide exchange factor
HDAC6: histone deacetylase 6
Hh: hedgehog
HSC: hepatic stellate cells
INPP5E: phosphatidylinositol polyphosphate 5-phosphatase type IV
iPSC: induced pluripotent stem cell
MAPK: mitogen activated protein kinase
MEK: mitogen activated protein kinase kinase
mTORC1: mammalian target of rapamycin complex 1
NF1: neurofibromin 1
NPM1: nucleophosmin-1
PC: primary cilium
PDE6D: phosphodiesterase 6 δ subunit
PGK2: GMP-dependent protein kinase 2
PI3K: phosphoinositide 3-kinase
PIP_2_: phosphatidyl-inositol-4,5-bisphosphate
PS: phosphatidylserine
Shh: sonic hedgehog
SPRED1: sprouty related EVH1-domain containing protein 1
SREBP1: sterol regulatory element-binding protein 1
YAP: yes associated protein
